# Reverse the Lens, Set Focus on the Followers: A Theoretical Framework of Resource Dependence, Upward Influence, and Leadership

**DOI:** 10.3389/fpsyg.2021.699340

**Published:** 2021-09-24

**Authors:** Neha Tripathi

**Affiliations:** Department of Humans Resources Management, Indian Institute of Management Ahmedabad, Ahmedabad, India

**Keywords:** leadership, followership, upward influence, dependence relationship, power imbalance, joint dependence

## Abstract

Leadership theories predominantly focus on the top-down managerial influence on employees. Recent theoretical developments, however, have accentuated the call for scholarly attention on holistic models comprising both leadership and followership. In the present study, the author developed a theoretical framework of upward influence and leadership construction by drawing on resource dependence theory. Specifically, the author proposed a novel outlook illuminating upward influence in hierarchical relationships whereby employees, as the hosts of tacit resources, inculcate interdependent relationships with their managers. Considering the dependence of employees and managers on each other for tangible and intangible resources, relationships with a (a) power imbalance and (b) joint or embedded dependence emerge. The author further explained the role of leadership construction in power-imbalanced and embedded relationships and elaborated on organizational and team structural boundary conditions. By revitalizing upward influence, the proposed theoretical framework offered new insights into leadership and followership literature, with the potential to change the conversation from a foundational thesis assuming the managerial capacity to lead and bestow resources on their subordinates to a two-way resource-dependence perspective, which has been scarcely considered in contemporary management research.

Leadership is predominantly conceived as a top-down influence process that attracts profound scholarly attention in management research (Banks et al., 2018; Kark and Van Dijk, 2019). However, a plethora of leadership theories articulate the downward influence on subordinates, which is engendered by the personality and behaviors of leaders (Zaccaro, 2007; Bass and Bass, 2008; Hiller et al., 2011). Although this set of research has provided useful insights into managerial influence on subordinates, this narrow focus has unfortunately created a void in scholarship on upward leadership influence, to the extent that an inherent tendency to study leadership is conceived from studying top-down leadership (Uhl-Bien, 2006; Van Vugt et al., 2008). Thus, the present state of science remains limited in capacity, devoted to a scant focus on upward influence, and considered either in terms of influence tactics (Kipnis et al., 1980) or impression management (Gardner and Martinko, 1988) to gain tangible and intangible resources (Higgins et al., 2003), consequently ignoring the capacity of employees to influence higher authority from the perspective of an active source of leadership.

Recently, contemporary research has conceptualized leadership as “a broader, mutual influence process independent of any formal role or hierarchical structure and diffused among the members of a given social system” (DeRue and Ashford, 2010), articulating the plausibility of conduits of upward leadership coming from the subordinates to influence the superiors (Oc and Bashshur, 2013). Thus, upward influence and leadership construction in a supervisor–subordinate relationship bear importance from both practical and theoretical standpoints. From a practical standpoint, the thesis, employees are empowered with interpersonal influence on the supervisors, expresses cues to the organizations about the employees to be “an active source of leadership” rather “a passive recipient of leadership” traditionally viewed. Uhl-Bien et al. (2014) cautioned that organizations should rethink leadership, whereby “leaders and followers interact together in context to cocreate leadership and its outcomes,” thus acknowledging that employees, and not only managers, are active practitioners of leadership in organizations. From a theoretical standpoint, top-down leadership has been a celebrated topic in organizational behavior with its focal consideration devoted to the formally assigned hierarchical roles, where leadership originates from the leaders or formal supervisors and followership originates from the followers or formal subordinates. Within followership theories, such conventional views adhere to a role-based approach to leadership, in which followers in formal hierarchical roles (e.g., subordinates) are viewed as causal agents and leaders (i.e., managers) are seen as recipients or moderators of follower outcomes. However, a constructionist approach conceives leadership to be constructed in the form of relational interactions among people (DeRue and Ashford, 2010; Oc and Bashshur, 2013). Subordinates, along with superiors, are the active participants that co-construct the leadership, the followership, and the outcomes. From these insights, it becomes of utmost important to explain the personal and interpersonal attributes that contribute to upward leadership influence so that new theories advancing the scholarly understanding of socially co-constructed leadership can be developed.

By employing a resource-dependence lens (Emerson, 1962), in the present study, the author developed a theoretical framework illuminating upward influence and leadership construction in formal hierarchical relationships by considering employees as active sources of leadership. The conceptual model is presented in Figure 1. First, the personal attributes that leverage resource dependence and upward leadership influence were described. Although resource dependence lies at the center of leadership theories, thus far, extant theories have been built by assuming the dependence of employees on their managers. For instance, leader–member exchange theory (Foa and Foa, 2012) postulates the interdependent social exchange relationship between a manager and an employee; however, the contribution that employees give in return is considered intangible (e.g., trust, respect, and obligation) compared with the tangible resources from their managers (e.g., funds and information). In organizations, however, employees host resources in the form of human capital (Barney, 1991; Barney and Wright, 1998). A unique feature of human capital pertains to the inability of a firm to “own” this resource (Barney and Wright, 1998). In fact, a firm can only temporarily possess human capital resources through an employment agreement. Given that employees can leave the firm anytime, firms can possess, but not own, the human capital for a defined period. Tacit resources possessed by employees create dependence for the managers (Blau, 1955; Crozier, 1964). In the proposed theoretical framework, the author explained that the resources possessed within the personal capacity of employees can foster upward influence on higher authorities.

**Figure 1:**
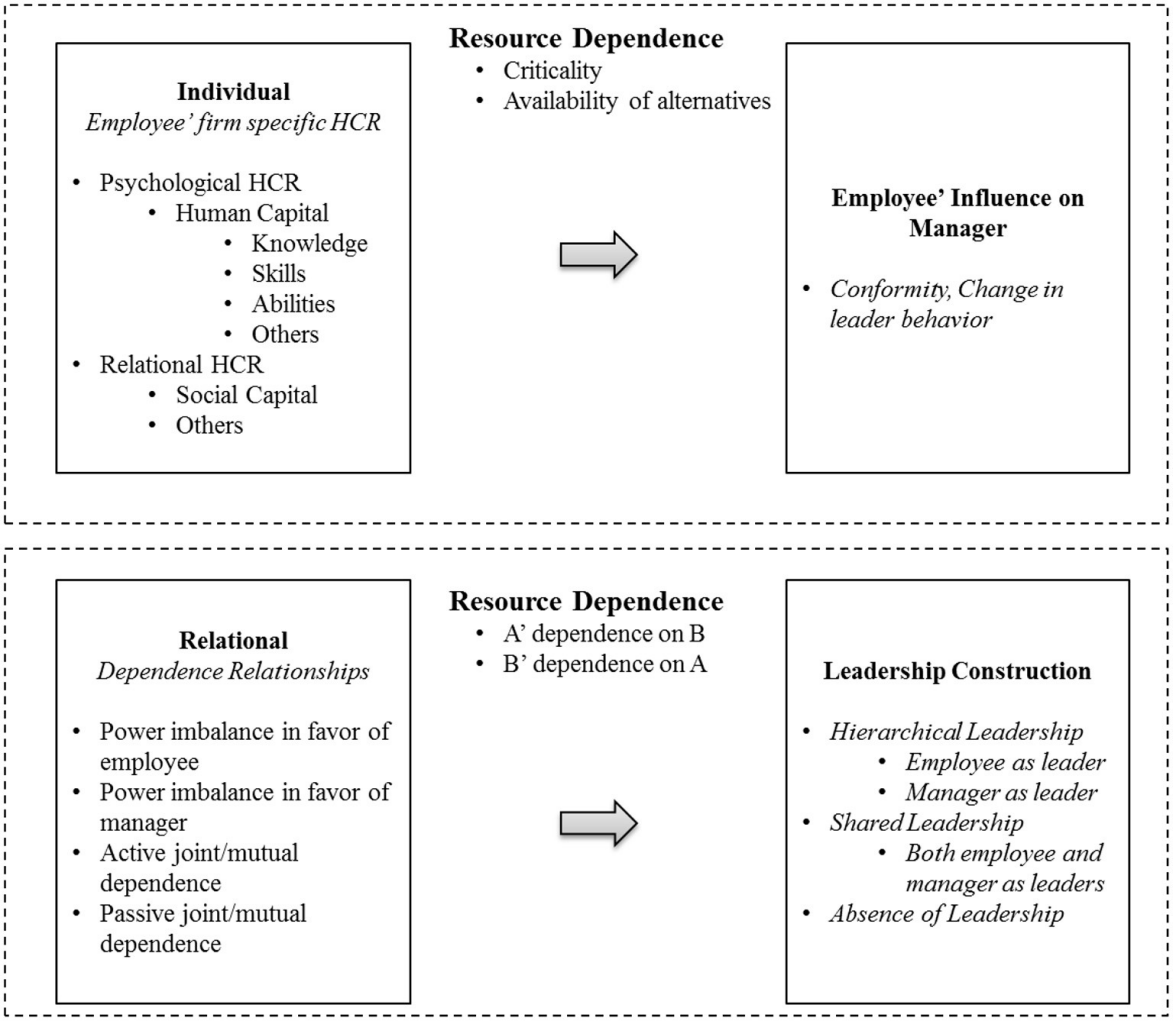
Employee influence on a manager: A resource-dependence perspective.

Second, the theoretical model explained the interpersonal attributes of dependence relationships and leadership construction. From an interpersonal perspective, influence is conceived of a property of the social relation (Emerson, 1962). Interpersonal influence among dyads requires the consideration of the influence capability of A in relation to B and the influence capability of B in relation to A, simultaneously. Furthermore, the interdependence between managers and employees is affected by both (a) the dependence of employees on their managers and (b) the dependence of the managers on their employees. Two types of dependence relationships, (a) asymmetric, referring to power imbalance, and (b) embedded, referring to joint/mutual dependence, thus emerge (Pfeffer, 1972; Pfeffer and Nowak, 1976; Provan et al., 1980). More recently, the power-dependence relationship between supervisor and their subordinates has gained scholarly attention (see Wee et al., 2017). However, the research, thus far, has devoted more attention to workplace mistreatment by managers (Wee et al., 2017) than providing a generic framework of leadership co-construction to advance the theory of upward influence that informs a knowledge base of a wider range of leadership constructs. The author, herein, explained how leadership is co-constructed in power-imbalanced and embedded relationships between employees and managers based on the leader identity claims (DeRue and Ashford, 2010) sought by the employees and whether these claims met the acceptance or refusal of their managers.

Third, leadership is a context-dependent phenomenon contingent on external factors such as team and organizational structures (Fiedler, 2015). Specifically, leader effectiveness manifests within the boundaries of team structures (Bird, 1977; Thamhain, 2004; Muethel and Hoegl, 2013; Greer et al., 2018; Koeslag-Kreunen et al., 2018). Furthermore, teams, both specialist and uniform, are the rudimentary building blocks of organizations. In particular, specialist teams comprise of team members who possess unique firm-specific tacit knowledge complementary to other team members, while uniform teams comprise of members who possess supplementary or equitable expertise. Likewise, organizations, both organic and mechanistic, differ in rules, routines, and formal structures (Burns and Stalker, 1961). Whereas, mechanistic organizations follow bureaucratic management by leveraging relatively stable and routinized work environment, organic organizations adapt to dynamism and instability by calling for agile management. These diverse work settings call for the scrutiny of boundary conditions when and how upward leadership influence is formulated in the supervisor–subordinate relationship.

The theoretical framework presented in this study contributed to literature in three important ways. First, the framework contributed to leadership literature. Leadership theories assume that leadership arises from a higher hierarchical figure to influence a person at a lower hierarchy. In these previous studies, the influence from a person from a lower hierarchy on someone from a higher hierarchy is often considered impression management, which ignores the social influence process embedded in a leader–follower framework (Oc and Bashshur, 2013; for an exception, see Wee et al., 2017). In response, the theoretical framework in the present study provided a guiding framework of upward influence, enhancing the scholarly understanding of leadership construction. Second, the framework contributed to followership literature. Although followership theories acknowledge followership along with leadership and study it in relation to leadership, this set of research has mainly elaborated on the characteristics, dispositions, and behaviors of followers that co-create leadership and followership (Uhl-Bien, 2006; Van Vugt et al., 2008; Oc and Bashshur, 2013; Uhl-Bien et al., 2014), which, in turn, ignores upward influence. Likewise, in the organizational behavior domain, asymmetric and joint dependence in interpersonal relationships focus on investigating power dynamics and its consequences (Thibaut and Kelley, 1959; Emerson, 1962, 1964; Kelley and Thibaut, 1978). Furthermore, the influence of employees on their managers has been studied in a limited capacity, specifically, in the context of “upward” influence tactics (Kipnis et al., 1980) to gain favorable work outcomes such as performance assessment, salaries, and promotions (Higgins et al., 2003). The research thus far has provided limited insight into upward influence in asymmetric and embedded dependence relationships, thus constructing leadership in the organizational hierarchy. In contrast, the present theoretical framework contributed to the existing literature by explaining manager–employee dependence and influence relationships directly by illuminating the personal and interpersonal attributes of upward influence and leadership co-creation through claims of leader identity.

## Theoretical Background

### Upward Influence and Leadership Construction

Interpersonal influence has been a topic of considerable scholarly interest among management scholars and practitioners alike. Interpersonal influence, which is the capacity to affect the psychological states, decision-making, or behaviors of others, is studied in a wide range of socio-organizational contexts, including power, politics, sales, marketing, and impression management (Bearden et al., 1989; Crosby et al., 1990; Brown and Moshavi, 2005). For instance, interpersonal power represents social influence as the ability of a person “to influence others and make them do things they would not do otherwise” (Sturm and Antonakis, 2015, p. 139), which consequently affects the thoughts, emotions, and actions of other individuals. Interpersonal influence from peers is also noteworthy in the context of organizational politics (Robinson, 1976). Consumer behavior and marketing research further show that consumers are susceptible to interpersonal influence by salesmen (Bearden et al., 1989). Interpersonal influence is effective in non-work situations, and not only in the work contexts, where friends and family members become important sources of influence in the lives of individuals and their career-oriented decisions (Werner-Wilson and Arbel, 2000; Fouad et al., 2010).

More recently, scholars have accentuated the need to utilize a social influence lens to argue for leadership construction within a social influence ecosystem, where a person interacts with others (Graen and Uhl-Bien, 1995; DeRue and Ashford, 2010) with the capacity to affect their behaviors (Brown and Moshavi, 2005). The social environments of people (i.e., closeness, status, and number of people as sources of impact) promote influence in social settings (Latane, 1981, 1996). Furthermore, leadership as social influence is constructed in interpersonal relationships when one member “claims leader or follower identity” and another member “grants leader or follower identity,” thus facilitating an interpersonal social context by creating and recreating such identities (DeRue and Ashford, 2010: 631). In this process, interpersonal influence, power, and dependence are closely interrelated. Considering leadership construction (DeRue and Ashford, 2010) from a resource dependence (Emerson, 1962) lens, the author describes (a) the personal and (b) the interpersonal attributes of upward influence in the next sections.

## Theory and Propositions

### Personal Attributes, Resource Dependence, and Upward Influence

Resource dependence theory (Emerson, 1962) argues that the influence of person A on person B underlies the dependence of B on A for some kinds of resources. For the most part, managers are considered to possess tangible resources for the employees in a typical Weberian organizational setting (Weber, 1947, 1982), which remains a central assumption in relational leadership theories (e.g., leader-member exchange theory, Graen and Uhl-Bien, 1995). More recently, to unpack the influence of employees on their managers and dependence of managers on employees, scholars have identified the characteristics and behaviors of followers that have been found to influence and cause a change in leader behaviors (Oc and Bashshur, 2013; Wee et al., 2017). In the increasingly flattening work environment of today, managers depend on employees to fulfill work needs/goals and gain important tacit information (e.g., Lussier and Achua, 2007). Thus, human capital resources encompassed by the employees enable the dependence of managers on their employees.

According to the resource-based theory (Barney, 1991), human capital resources are sources of sustainable competitive advantages for a firm (Barney and Wright, 1998; Kraaijenbrink, 2011). The effect of human capital resources has been widely studied in literature on strategy and strategic HR at the individual, team, and organization levels (for a review, see Ployhart et al., 2014). At the individual level, human capital resources (HCR) are categorized in two forms, (a) psychological HCR and (b) relational HCR (Ployhart et al., 2014). First, the psychological approach to HCR proposes knowledge, skills, ability, and other characteristics (KSAOs) as the underlying components of HCR. Noe et al. (2006) and Schmitt and Chan (1998) defined individual-level KSAOs, where *knowledge* referred to the factual or procedural information necessary for performing a specific job; *skills* referred to the level of proficiency of an individual and their capabilities to perform a specific job task; *ability* referred to the more enduring capability (usually cognitive) that is necessary for an individual to perform a job; *other characteristics* referred to personality traits or other attributes that affected the ability of individuals to perform a specific job. For instance, in the classic conceptualization of human capital, Becker and Gerhart (1996) noted that human capital encompassed a greater array of aspects, including behaviors, stating that “the concept of human capital also covers accumulated work and other habits... The various kinds of behavior included under the rubric of human capital help explain why the concept is so powerful and useful” (Becker and Gerhart, 1996, p. 9–10).

Second, relational human capital resources refer to the social capital of an individual as a socially derived building block of the human capital (Coleman, 1988). Although social capital literature has evolved separately from human capital literature, such that social capital scholars have looked upon the “structural” and “network” aspects of human relationships, whereas human capital scholars have looked upon the “cognitive” and “psychological” aspects of human abilities, there exists work within broader literature that explicitly considers the interrelation of human and social capitals (Nahapiet and Ghoshal, 1998; Adler and Kwon, 2002). Scholars have conceptualized social capital as a property or attribute of individuals (Bourdieu, 1986; Sobel, 2002) where the value embedded in the relationships of employees is considered a resource (i.e., much like their skills and abilities) and that the employees can use such relational human capital to benefit the organization. Drawing from these insights, the present article considered the social capital of individuals as a socially derived building block of the HCR; thus, a relational HCR.

Human capital resources can be conceived as generic or firm-specific depending on the generalizability of these resources across organizations (Wright and McMahan, 2011). Becker and Gerhart (1996) defined firm-specific HCR to be useful only in the firm employing the individuals, whereas generic HCR are useful in other firms. For instance, firm-specific psychological KSAOs may include tacit knowledge of the procedures of the firm and special certifications that are required to perform work-related roles.

Likewise, specific relational human capital refers to the valuable relationships/ties an employee has with people or other parties who own resources or power that have a proximal or direct impact on the performance and goals of the individual, team/s, or organization where the employee works. On the other hand, generic KSAOs may include the education level and skills useful for the employability of an individual across organizations, while the generic relational HCR refer to the possession of employees with valuable relationships/ties with people or other parties who possess resources or power that have a distal or indirect effect on their in- and extra-role performance or on the future career aspirations and goals of the individuals in general.

According to the resource-dependence theory (Emerson, 1962; Pfeffer and Salancik, 1978), actor A will be dependent on actor B if actor B possesses some resources that are needed by actor A. In short, influence is reciprocal of dependence; that is to say that the actor B will have more influence on actor A if actor B holds the resources that are needed by actor A. Translating this resource-dependence logic into a manager–employee relationship suggests that an employee will have a higher influence on their manager in case the employee possesses some resources that are needed by the manager. In other words, the manager will be dependent on the employees who possess more firm-specific HCR than generic HCR. Thus, the author posited that the firm-specific human capital of an employee will positively relate to the influence of that employee on his/her manager. Accordingly,

*Proposition 1: Firm-specific human capital resources of an employee will positively relate to their influence on a manager*.

#### Criticality and Availability of Alternatives

Resource-dependence theory (Emerson, 1962; Pfeffer and Salancik, 1978) further argues that dependence is a function of resource criticality and the availability of alternative providers of critical resources. An actor A, therefore, is dependent upon actor B (1) in proportion to the need A has for resources that B can provide and (2) in inverse proportion to the availability of alternative actors capable of providing the same resources for A. Translating this dependence relationship into the manager–employee relationship suggests that a manager is dependent on an employee (1) in proportion to the need of that manager for resources that the employee can provide and (2) inversely with the availability of alternative employees capable of providing the same resources for the manager. Thus, the author posits,

*Proposition 2: Criticality and the lack of available alternatives strengthen the positive relationship between firm-specific human capital resources of an employee and their influence on a manager*.

### Interpersonal Attributes, Resource Dependence, and Upward Influence

Dependence relationships emerge as an employee and a manager interact for exchanges of tangible and intangible resources. Dependence relationships are bidirectional; that is, the interdependence between actor A and actor B not only refers to the dependence of A on B but also to the dependence of B on A. An actor A is dependent upon actor B (1) in proportion to the need of A for resources that B can provide and (2) in inverse proportion to the availability of alternative actors capable of providing the same resources for A. Conversely, actor B is dependent upon actor A (1) in proportion to the need B has for resources that A can provide and (2) in inverse proportion to the availability of alternative actors capable of providing the same resources for B. Translating this to manager–employee relationship, the interdependence of a manager and an employee will be affected by both (a) the dependence of the manager on an employee and (b) the dependence of the employee on the manager. An employee is dependent on the manager (1) in proportion to the need of the employee for resources that the manager can provide and (2) inversely with the availability of alternative managers capable of providing the same (or better) resources for the employee.

The concept of interdependence has received considerable attention from scholars studying interorganizational relations. Scholars have suggested two types of interdependence: “asymmetric” and “joint.” Most of the early research on organizations considered the interdependence between actors to be asymmetric and a liability that needed to be managed (Pfeffer, 1972; Pfeffer and Nowak, 1976; Provan et al., 1980). The unequal dependence would cause a power imbalance that is likely to be detrimental for the weaker actor (Thompson, 1967). Emerson (1962) distinguished between joint dependence in a dyad, or the *sum* of dependencies of actors on each other, and dependence asymmetry, or the *difference* in dependencies of actors on each other. Casciaro and Piskorski (2005) termed these two distinct theoretical dimensions of resource dependence as *mutual dependence* and *power imbalance*.

**Figure 2:**
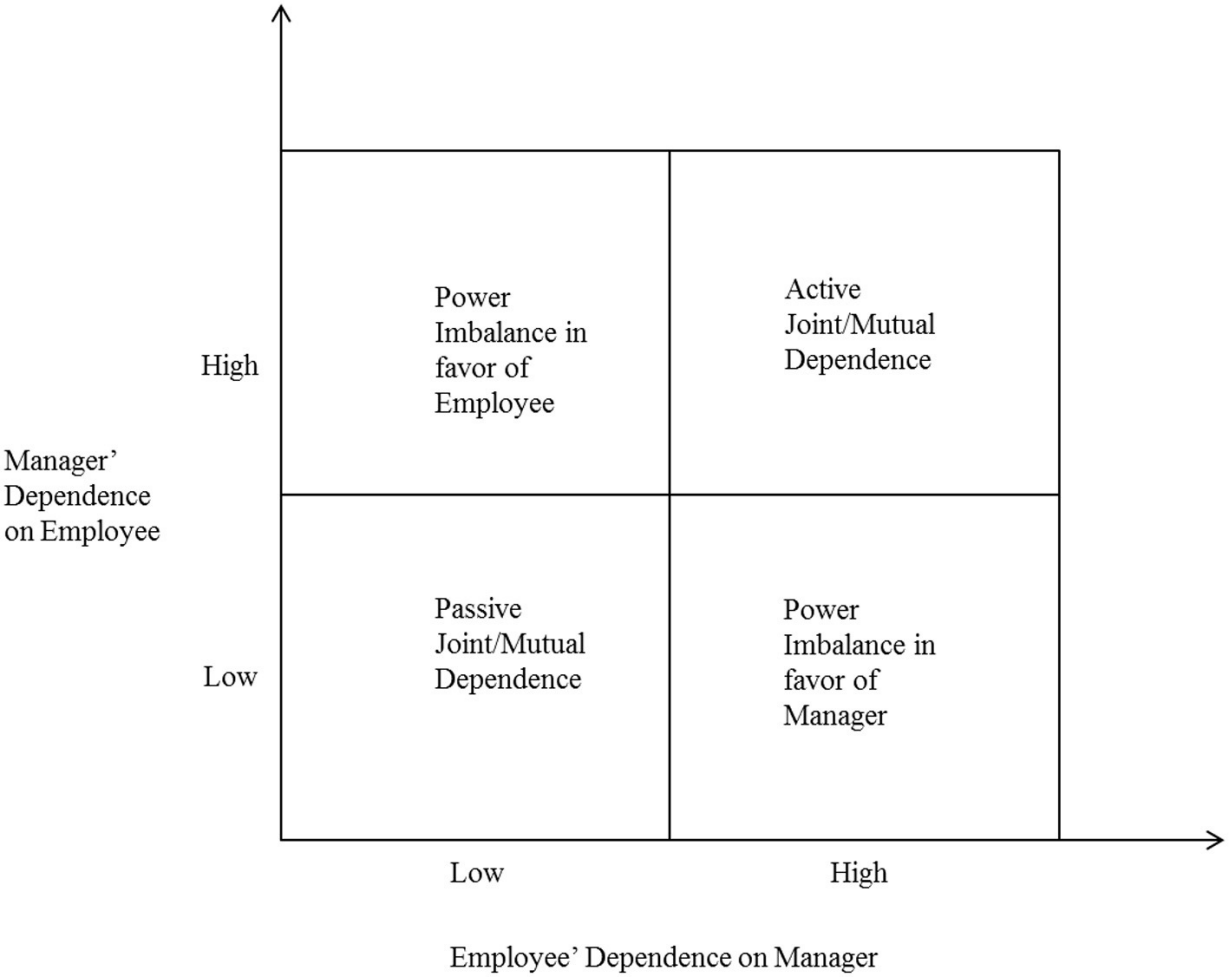
Dependence relationships between an employee and a manager.

Mutual dependence captures the existence of the bilateral dependencies in a dyad, regardless of whether the dependencies of two actors are balanced or imbalanced (Gulati and Sytch, 2007). Gulati and Sytch (2007) found that joint/mutual dependence (or embeddedness) led to higher performance, such that firms were more likely to use joint actions, show higher trust, and exchange more information. Formally, this conceptualization is defined in terms of the sum or the average of the dependence of actor A on actor B and the dependence of actor B on actor A. Mutual dependence arises in employee–manager relationships when (a) the manager is highly dependent on the employee and the employee is also highly dependent on the manager, which means there is active joint/mutual dependence, or (b) neither the manager is dependent on the employee nor is the employee dependent on the manager, which means there is passive joint/mutual dependence (See Figure 2).

Power imbalance captures the difference in power of each actor over the other. Formally, this construct can be defined as the difference of dependencies between two actors or the ratio of the power of the more powerful actor to that of the less powerful actor (Pfeffer, 1972; Pfeffer and Nowak, 1976; Lawler and Yoon, 1996). Power imbalance arises in employee–manager relationship when (a) the manager is highly dependent on the employee but the employee is not highly dependent on the manager, that is, the power imbalance is in favor of the employee, or (b) the employee is highly dependent on the manager but the manager is not highly dependent on the employee, that is, the power imbalance is in favor of the manager. Overall, as depicted in Figure 2, the interdependence of a manager and an employee creates four kinds of dependence relationships, (a) power imbalance in favor of an employee, (b) power imbalance in favor of a manager, (c) active joint/mutual dependence, and (d) passive joint/mutual dependence.

#### Leadership Construction: Power Imbalance in Favor of an Employee

Power imbalance in favor of an employee refers to the dependence relationship that emerges when employees hold critical tangible and intangible resources needed by a manager and the manager lacks alternatives. Numerous organizational contexts host such relationships. For instance, the relationship between a software architect (technical subject matter expert) and a project manager in a software development team can create a power imbalance in favor of the technical expert employee. A chief architect is an expert in software design responsible for design choices, standards, coding, tools, and platforms. A project manager, on the other hand, is responsible for the successful execution of a project within the given time and approves the budget-fulfilling requirements of stakeholders. In most organizations, career paths for an architect and a manager delineate in two parallel career ladders. Although a software architect may formally report to the project manager, as a subject matter expert, they possess tacit knowledge and information. Thus, the chief architect who is considered valuable and difficult to replace with generic alternate choices holds considerable power and dependency to influence the manager. In his classic work, Crozier (1964) explained such a relationship as hosted between maintenance workers and supervisors. The maintenance workers kept tacit knowledge, blueprints, and maintenance directions exclusive to themselves to preserve control over machines and tools. The maintenance workers constantly attacked supervisors, making it impossible for them to assert their authority (Crozier, 1964: 154). Crozier (1964) highlighted that, surprisingly, rather than picking a fight over authority with maintenance workers, the supervisor took an “adjustment-like” approach by lowering their involvement and participation.

**Figure 3:**
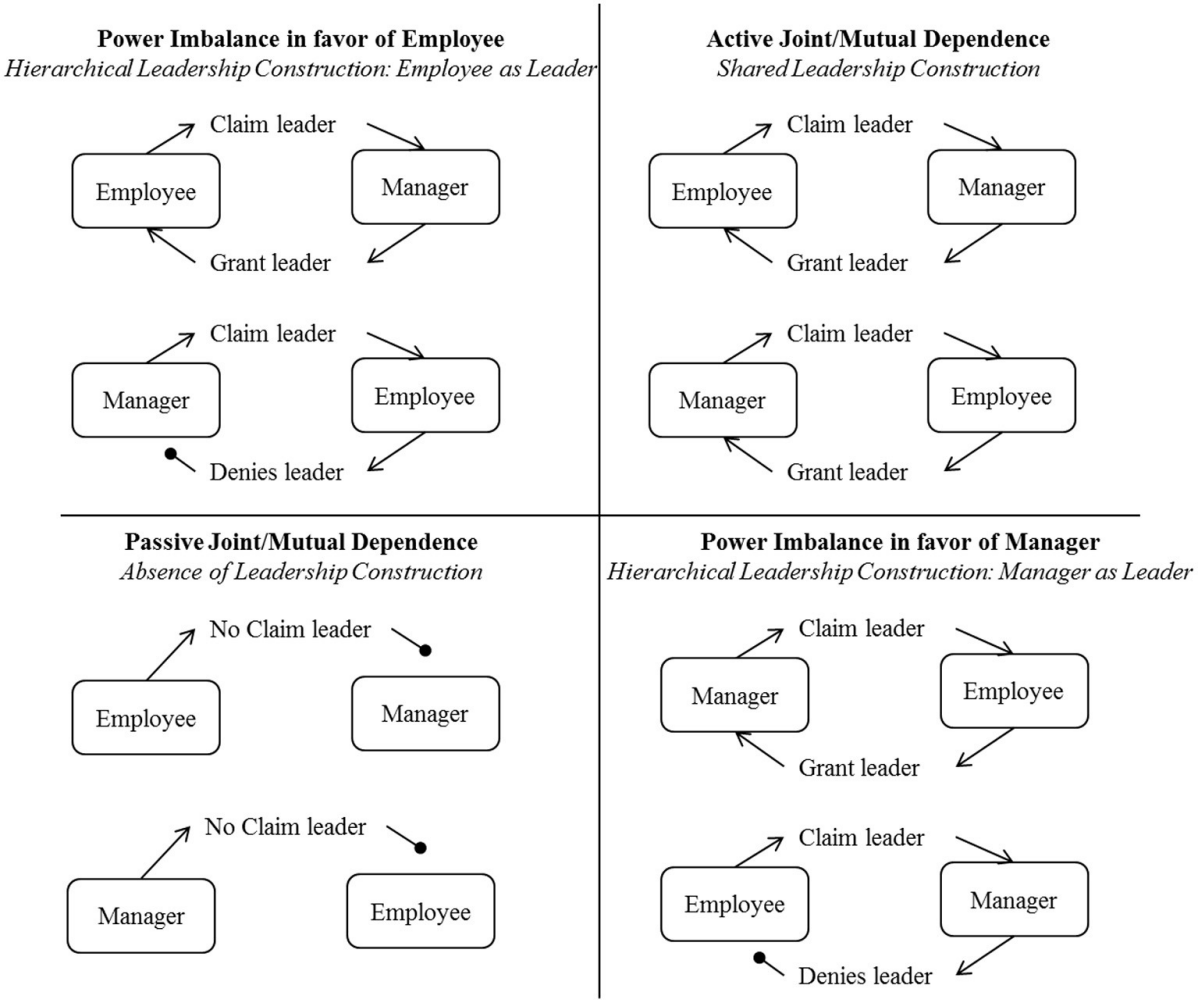
Leadership construction in dependence relationships.

Upward leadership is constructed in such dependence relationships when an employee claims to be the leader and a manager grants leadership, nodding in acceptance of the informal leadership of the employee. Extant research suggests that, when the leaders lack the expertise to lead, the employees use upward influence by means of legitimization and direct pressure on the leaders (Cable and Judge, 2003). Kipnis and Schmidt (1983) reported that subordinates sought to establish the legitimacy of a request either by claiming the authority to make it or by verifying that it is consistent with organizational policies, rules, practices, or traditions. For instance, people use assertiveness, bargain to put pressure, and make use of demands and persistent reminders to influence others (Kipnis and Schmidt, 1988). Crozier (1964) noted that the subordinates holding informal but powerful positions with respect to a superior showed authoritative and directive behaviors, including assertion, discipline, and submission from others, as quoted, “the one unforgettable sin of a machine operator is to “fool around” with his or her machine” (p. 153). In such scenarios, employees claim the leader identity, and since these employees hold tacit resources, the manager is likely to grant them the leader identity, acknowledging their competence and worthiness to take on the leadership roles (Figure 3). Accordingly,

*Proposition 3: When a manager and an employee have a dependence relationship with a power imbalance in favor of the employee, (a) the attempt of the employee to claim leadership is granted by the manager and (b) the attempt of the manager to claim leadership is denied by the employee*.

#### Leadership Construction: Power Imbalance in Favor of a Manager

The power imbalance relationship in favor of a manager is the most studied dependence relationship between employees and managers. In a typical bureaucratic organization, employees possess little organizational power, i.e., little control over budgets, policy, or personnel matters, and have few personal or organizational objectives that require compliance from others. Ansari and Kapoor (1987) found that individuals were more likely to employ ingratiation when their target was authoritarian. Similarly, Farmer et al. (1997) found that “soft” influence tactics were related to a locus of control and self-monitoring, for example, seeking to get a target in a good mood or to think favorably of the sender before asking the target to do something or appealing to the feelings of loyalty and friendship of the target before asking them to do something. In particular, employees used upward influence *via* consultation with charismatic and inspirational leaders (Cable and Judge, 2003). These “bystander” employees used little influence with their superiors (Kipnis and Schmidt, 1988).

Accordingly, the author posited that, when employees and the manager are bonded with a power imbalance in favor of the manager, the employees do not actively attempt to claim a leader identity. In any instances when employees claim leader identity, the manager is likely to deny such attempts since such employees are perceived to lack in tacit resources and the adequate competence/power to lead. For example, Crozier (1964) noted that, while supervisors are likely to accept influence from maintenance workers who possessed tacit knowledge of machines, supervisors denied any such attempt by production workers who lacked this tacit knowledge. Accordingly, the author posits,

*Proposition 4: When a manager and an employee have dependence relationship as power imbalance in favor of the manager, (a) attempt of the manager to claim leadership is granted by the employee, and (b) attempt of the employee to claim leadership is denied by the manager*.

#### Leadership Construction: Active Joint Dependence

Active joint/mutual dependence relationships form when both the employee and the manager possess critical resources needed by the other. Unlike the hierarchical leadership seen in power-imbalanced relationships, a participative and shared leadership is likely evolved between the employee and the manager in embedded relationships. Gulati and Sytch (2007) argue that embeddedness promotes joint actions, higher levels of trust, and the exchange of information between the engaged entities. Participative or shared leadership implies that the leaders and their employees consult, ask for suggestions, and take collective ideas into consideration before making decisions (Chen and Tjosvold, 2006; de Poel et al., 2014).

Ansari and Kapoor (1987) highlighted that individuals were more likely to use rational persuasion, logical arguments, and factual evidence toward the attainment of task objectives when their target was participative. “Tactician” employees used an average amount of influence and emphasized reason. Thus, it is likely that the employee and the manager are both open to claim and grant leader identities to each other. Tactician managers directed organizational subunits involved in non-routine work, which had been found in other settings (Salancik and Pfeffer, 1977), and provided them with a skill and a knowledge power base. Tacticians relied on reason and logic to gain compliance. First, the employee and the manager engage in *consultation*, which refers to the seeking of joint participation in planning a strategy, activity, or change for which the assistance of the other is desired. Second, they participate in *exchange*, which refers to the offering of an exchange of favors with a target that indicates a willingness to reciprocate at a later time and a promise to share the benefits if the target helps. Last, the parties form a *coalition*, which refers to the seeking of aid from others to persuade a target to do something or using the support of others as a reason for the target to agree. Accordingly, the author posits,

*Proposition 5: When a manager and an employee have a dependence relationship with an active joint/mutual dependence, (a) the attempt of an employee to claim leadership is granted by the manager and (b) the attempt of a manager to claim leadership is granted by the employee*.

#### Leadership Construction: Passive Joint/Mutual Dependence

Passive joint dependence relationships are formed when neither an employee nor a manager possesses the critical resources needed by the other. A passive joint/mutual dependent relationship is likely to stay dormant unless external organizational forces build an “interpretive context” that increases the salience of dependence between the manager and the employee. Accordingly, the author posits that neither employee nor the manager claims leader identity from the other,

*Proposition 6: When a manager and an employee have a dependence relationship with a passive joint/mutual dependence, neither (a) the employee attempts to claim leadership nor (b) the manager*.

### Contingencies: Team and Organizational Structures

Social influence and leadership construction are affected by situational contingencies such as team and organizational structures (Klein et al., 2006; Day et al., 2014; Zhu et al., 2018). Teams are the rudimentary building blocks of an organization. Work teams, both specialist and uniform, differ in structure and composition. Specialist teams comprise team members who hold more unique firm-specific HCR than other team members, for instance, a team comprised of subject matter experts (SMEs, Klein et al., 2006). On the other hand, a uniform team comprises of members who have equitable firm-specific HCR. Resource-dependence theory (Emerson, 1962; Pfeffer and Salancik, 1978) argues that dependence is a function of the availability of alternative providers of critical resources. Actor A, therefore, is dependent upon actor B (1) in proportion to the need of A for resources that B can provide and (2) in inverse proportion to the availability of alternative actors capable of providing the same resources for A. Translating this dependence relationship into the manager–employee relationship suggests that a manager is dependent on an employee (1) in proportion to the need of a manager for resources that an employee can provide and (2) inversely with the availability of alternative employees capable of providing the same resources for the manager. In contrast, uniform teams facilitate more alternatives for the managers. Since the manager has a higher number of alternatives, it is easy for the manager to obtain resources from other employees in uniform teams as compared with specialist teams. Therefore, the author posits,

*Proposition 7: The positive relationship between the firm-specific human capital resources of an employee and their influence on a manager will be stronger in specialist teams as compared with uniform teams*.

Organizational structures influence leadership emergence and effectiveness (Stogdill and Shartle, 1948; Bass and Avolio, 1994). Organizations, both organic and mechanistic, differ in rules, routines, and formal structures (Burns and Stalker, 1961; Cruz and Camps, 2003). Mechanistic organizations follow bureaucratic rules and work more under relatively stable conditions compared with organic organizations. In the mechanistic systems, emphasis is given to the specialization of individuals through the promotion of independence to work separately and specializing in one type of task/s^1^. in this system, the hierarchy of formal authority is well-defined by an organizational chart and decision-making is centralized and vertical. The technical methods, duties, and powers attached to each functional role are precisely defined. Operations and work-related roles are governed by clear instructions and the decisions issued by the superiors.

On the other hand, organic organizations are adapted to internal or external instabilities, where the problems or the requirements for actions cannot be broken down and distributed among specialist roles within a clearly defined hierarchy. Individuals perform their work-related tasks in correspondence with the tasks of the firm as a whole. Jobs do not hold much formal definition in terms of methods, duties, and powers. Job roles and activities are continuously redefined by interactions with others. These interactions run both ways, laterally as much as vertically; consequently, the communication between people of different ranks tends to resemble lateral consultations rather than hierarchical.

Given that mechanistic organizations follow strict formal authority, managers have a higher influence on their employees by the virtue of the legitimate, reward, and coercive powers provided by organizations for the manager (French and Raven, 1959). On the other hand, organic organizations develop implicit norms and informal structures such that hierarchical relationships are diffused into lateral relationships. Therefore, the influence of employees on the manager due to resource dependence is offset by the dependence of the employee on the manager, owing to the legitimate power of the manager in mechanistic organizations. On the other hand, the influence of the employees on the manager due to resource dependence will be more evident in organic organizations because managers do not use bureaucratic hierarchical power on their employees. Accordingly, the author posits that organizational structures will moderate the effect of tacit HCR on influence on the manager. This effect will be stronger in organic organizations as compared with mechanistic organizations.

*Proposition 8: The positive relationship between firm-specific human capital resources of an employee and their influence on a manager will be higher in organic organizations as compared with mechanistic organizations*.

#### Joint Effect of Team and Organizational Structures

The mutual dependence and power-imbalanced relationships among employee–manager dyads are shown in the 2 (specialist vs. uniform team) × 2 (organic vs. mechanistic organizations) matrix in Table 1. An organic organizational structure facilitates an internal environment conducive to the influence of an employee on the managers due to the dependence of the manager on the HCR of the employee. In such a (organic) favorable environment, specialist teams will further strengthen the positive effect of the firm-specific HCR of the employee on the manager (Proposition 2). The firm-specific HCR of the employee will have the strongest association with the influence an employee has on the manager in specialist teams in organic organizations, as a power imbalance emerges in favor of the employee. The positive effect of a (organic) favorable environment is buffered in uniform teams because alternatives are available for the managers within the team. Thus, in organic-uniform teams, the employee and the manager remain mutually dependent and build joint dependent relationships by instilling trust, promoting joint actions, and sharing information (Gulati and Sytch, 2007). In all, the author posits,

**Table 1:** Dependence relationships: Contingencies—team and organizational structures.

		**Manager's dependence on Employee (Team Structure)**	
		**High (Specialist Team)**	**Low (Uniform Team)**
Employee's Dependence on Manager (Organizational Structure)	Low (Organic)	*Power imbalance in favor of employee*	*Active joint/mutual dependence*
	High (Mechanistic)	*Active joint/mutual dependence*	*Power imbalance in favor of manager*

*Proposition 9: In organic organizations, the manager–employee relationship will be embedded–jointly dependent in uniform teams and asymmetric in specialist teams, such that the positive relationship between the firm-specific human capital resources of an employee and their influence on a manager will be strongest in specialist teams as compared with uniform teams*.

On the other hand, a mechanistic organizational structure creates an internal environment, which restricts the influence of an employee on managers due to the power of the manager over the human capital resources of the employee because of bureaucratic rules of authority and conformity (Weber, 1982). In such a (mechanistic) strict hierarchical environment, the uniform teams will enable managers to choose from alternatives, which will further restrict the influence of the employee on the manager. Thus, the firm-specific HCR of the employee will have the weakest association with the influence of the employee on the manager in uniform teams in mechanistic organizations, as a power imbalance exists in favor of the manager. The negative effect of a (mechanistic) restrictive environment is balanced in the specialist teams because managers will not have many (or any) alternatives available within their teams and will consequently need to rely on their employees for this tacit HCR. Thus, in mechanistic-specialist teams, the employees and the managers are mutually dependent whereby managerial influence arises due to bureaucratic structures, while the influence of an employee arises due to his/her expertise in the job roles^2^. Therefore, the author posits,

*Proposition 10: In mechanistic organizations, the manager–employee relationship will be embedded–jointly dependent on specialist teams and asymmetric in uniform teams, such that the positive relationship between the firm-specific human capital resources of an employee and their influence on a manager will be weakest in uniform teams as compared with specialist teams*.

## Discussion

The theoretical framework explained upward influence and leadership construction processes from a resource-dependence lens by considering employees as active sources of leadership. The framework elaborated on the personal and interpersonal attributes of upward influence whereby employees create dependence and thus influence the managers. On this basis, in the interpersonal context, employees act to claim leadership identity, which meets either the acceptance or refusal of the managers contingent on the team and organizational boundary conditions. In particular, the framework discussed upward influence in specialist vs. uniform teams and mechanistic vs. organic organizations. Whereas, specialist teams facilitate opportunities to claim leader identity so that employees can influence higher authorities through dependence on the tacit HCR, uniform teams provide more alternatives for managers, consequently reducing dependence on the employees and, in turn, restricting the opportunities of employees to claim leadership from higher authorities. Lastly, the framework illustrated the moderating effect of mechanistic vs. organic organizational structures. In mechanistic organizations, the hierarchical structure is stable and well-defined, giving rise to the traditional top-down leadership. However, in organic organizations, lateral and relational relationships flourish, providing employees the openness to influence higher authorities. Overall, as proposed in the theoretical framework, two kinds of dependence relationships, power-imbalanced and embedded, emerge. Upward leadership influence is co-created by employees and managers in power-imbalanced and embedded relationships through the claims of employees of the leader identity.

### Theoretical Implications

The proposed theoretical framework contributed to the literature in several important ways. First, the extant leadership literature abounds in leadership theories focusing on “leading” (Zaccaro, 2007; Bass and Bass, 2008; Hiller et al., 2011). The tremendous focus on “leading,” however, has unfortunately undermined the importance and usefulness of “following,” so much so that “following” has been considered less than desirable. Could there be a leader without a follower? To make the matter worse, these leadership theories ignored the active role of the followers, as only a handful of leadership models included followership (Uhl-Bien, 2006; Uhl-Bien et al., 2014). On the other hand, followership theories have mainly focused on investigating the desirable attributes of followers (Oc and Bashshur, 2013). Both the existing leadership and followership theories assumed that leadership arises from a higher hierarchical figure to influence a person at a lower hierarchy. The influence of a person from a lower hierarchy on someone from a higher hierarchy is often considered impression management, which ignores the social influence process embedded in a leader–follower frame. Although recent research has shown empirical evidence for a two-way dependency between managers and subordinates (Wee et al., 2017), the research is limited within the context of abusive supervision and workplace mistreatment. The theoretical framework in the present study contributed to leadership and followership literature by providing a guiding framework of upward influence that enhances the scholarly understanding of leadership construction from a generic perspective applicable and is relevant to a wider range of leadership constructs.

Second, resource dependence theories explain interdependence relationships between manager –employee dyads, especially in investigating the influence that rises from the bottom-up. Scholars in sociology and social psychology were among the first to theorize asymmetric and join dependence in interpersonal relationships (Thibaut and Kelley, 1959; Emerson, 1962, 1964; Kelley and Thibaut, 1978). However, in the organizational behavioral context, such dependence relationships were explored to examine power dynamics and their consequences, including punitive/coercive actions or strategies, conflicts, and constraint absorption (Gundlach and Cadotte, 1994; Kumar et al., 1998; Casciaro and Piskorski, 2005; Gulati and Sytch, 2007). Although the classic ethnographic studies by scholars like Blau (1955); Crozier (1964), and Gouldner, 1954 provided insights into superiors being influenced by employees through the formation of informal structures and relationships that emerged in the process of institutionalization as organizations adapted to external and internal environmental contingencies, such upward influence and dependence relationships were not investigated in greater depth afterwards. Therefore, studying the dependence of managers on employees and, thus, the influence of employees on managers sheds light on the influence process that rises from the bottom to the top, instead of flowing top to the bottom.

Third, the influence of employees on their managers had been studied in a limited capacity, as they elaborate “upward” influence tactics (Kipnis et al., 1980) to gain favorable work outcomes, including performance assessment, salaries, and promotions (see meta-analysis, Higgins et al., 2003). However, this stream of research mainly examined how the influence tactics of employees help them gain extrinsic success (Gardner and Martinko, 1988; Higgins et al., 2003) and not form leadership *per se*. Yukl and Falbe (1990) found that people use differential influence tactics in attempts to exert upward, downward, or lateral influences. Whereas, downward influence attempts were abundant with pressure, consultations, and inspirational appeals, upward influence was limited to requests for resources, approvals, and support. Essentially, followers were depicted not to “influence,” but as “influenced by” the higher authorities.

In their earlier work, Perreault and Miles (1978) identified combinations of influence strategies; later, Kipnis and Schmidt (1988) suggested upward-influence styles. People used inspirational appeal, ingratiation, and pressure in downward influence; personal appeal, exchange, coalitions, rational persuasion, and legitimation were used mostly in lateral and upward influences (Kipnis and Vanderveer, 1971; Yukl and Tracey, 1992). Extant research has suggested that downward influence styles are detrimental if used in the context of upward influence. For instance, male subordinates using a “shotgun” style of upward influence (i.e., emphasizing on assertiveness and bargaining) were evaluated less favorably by their superiors, earned less, and reported more work-related tensions and personal stress. In contrast, a logical and reason-based “tactician” approach to influencing superiors yielded more favorable individual outcomes. However, this stream of research provides limited insight into the influence styles that emerge in asymmetric and embedded dependence relationships. Thus, the proposed framework contributed to the literature by illuminating upward influence in asymmetric and embedded dependence relationships and elaborating on team and organizational boundary conditions.

Lastly, the proposed framework also contributed to the research on human capital. In their review on human capital research, Ployhart et al. (2014) highlighted that human capital was a multilevel construct studied by both strategy and HR researchers alike. While strategy researchers studied human capital as the HCR of a firm (Coff and Kryscynski, 2011), strategic human resources management (HRM) researchers explored HCR as a mediator between human resources policies and unit^3^ performance (Wayne et al., 1997; Wright and McMahan, 2011). At the individual level, either the HCR (e.g., education, training, etc.) predicted performance of an employee or the HR policies predicted the performance of a firm, which is often criticized for creating a “black box” for how the HCR of employees can influence managers, teams, and the organization above and beyond personal-level outcomes. The present study contributed to the existing literature by opening the black box and exploring the manager–employee dependence and influence relationships directly through the investigation of the impact of the HCR of employees on managers (unlike the existing focus on the consequences of employees such as work-related performance, Ployhart et al., 2014).

### Practical Implications

Leadership, dependence, and resources are critical concepts that bear practical relevance to managers and organizations. The present research recommends that organizations consider managers and employees beyond formal structural perspectives (i.e., hierarchical positions in the organization) as a function of interrelational resource dependence. Two meaningful messages evolved from the proposed framework. First, the co-construction of leadership employs active participation from both leaders and followers from a resource-dependence barometer to forecast upward influence. The scholarship of resource dependence in a leader–follower dyad empowers followers-centric notions to determine the perspective when leadership influence reverses the flow cascading up through the hierarchy. Organizations should be mindful of the social influence dynamics in the dyads by paying caution to how they may assume traditional viewpoints of leadership, which could be polarized toward the employees in certain work contexts, teams, or organizations. Second, managers should change their fixed mindsets to foster the leadership capabilities of followers by leveraging a work environment that is conducive to follower-centric leadership through positive interactions and the reduced use of denial when the followers attempt to claim the leader identity. More importantly, both leaders and followers must thoughtfully devise stability and congeniality in the dyadic relationship by paying attention to the nuances of resource dependence. In this regard, organizations should encourage norms and practices that their managers and employees can use to work together toward a mutual understanding and openness to recognize the bidirectionality, both upward and downward, of dependence, social influence, and leadership.

### Methodologies for Testability

The operationalization of firm-specific and generic human resources capital is of prime significance to empirically test upward influence and leadership construction in the proposed theoretical framework. The author suggests a few direct and indirect ways to operationalize this key construct. In a direct method, the researchers can collect useful information from job descriptions, open- and/or closed-ended questionnaires, and interviews with employees or other raters, for instance, supervisors. In an indirect method, the supervisory ratings of a person–job fit for an employee or the self-ratings of a person–organization fit can provide proxies for people to hold firm-specific human resources capital. Theories that have dominated a set of person–environment fit research in organizational psychology include the theory of work adjustment (TWA) (Dawis and Lofquist, 1984) and the attraction-selection-attrition (ASA) framework (Schneider et al., 1995), which capture various aspects of person–environment fit including, person–job fit (PJ fit), person–organization fit (PO fit), and person–team fit (PT fit), to name a few. Lastly, the author also recommends employing job characteristic models, for instance, the work design questionnaire (WDQ, Morgeson and Humphrey, 2006) and its adaptations in a diverse set of industries to design an indirect measurement of generic and firm-specific HCR in alignment with the attributes of the employees, the job, the firm, and the industry in general. The scholars are also encouraged to use both survey-based and social network-based methodologies to measure the psychological and relational components of human resources capital, dependence, influence, and leadership (Burt, 1984, 2000; Hoppe and Reinelt, 2010; Cullen-Lester et al., 2017; Peng et al., 2018).

### Future Research Directions

Leadership is a global cross-cultural concept. Growing interdependencies among nations have created the need to understand cultural differences pertaining to the concepts of leadership in different cultures. The cross-cultural understanding of leadership thus enhances our knowledge both of identifying the boundary conditions and understanding the universal aspects of leadership. The GLOBE research program (House et al., 2004) provides a substantial knowledge base on the cross-cultural dimensions relevant to leadership. The GLOBE study proposed wide variations in the values and practices relevant to the nine core dimensions of cultures. Thus, leadership is constructed within the boundaries of cultural dimensions, such as power distance, which is the degree to which members of an organization or society expect and agree that power should be stratified and concentrated at higher levels of an organization or government (Den Hartog et al., 1999; Resick et al., 2006). The author thus calls for future research to investigate the effect of cultural dimensions such as power distance on upward influence and leadership construction processes.

As Ayman and Korabik (2010, p. 159) eloquently put it, “A direct parallel exists between the dynamics that are due to culture and those that are due to gender. Both culture and gender have physical (visible) and value (invisible) components. Both affect identity and group cohesion, interpersonal interactions, and access to power and resources.” An extension to present a framework is to incorporate the interplay of gender and culture. According to the GLOBE study (House et al., 2004), countries differ in gender egalitarianism, which is the degree to which an organization or a society minimizes gender role differences while promoting gender equality. A diverse set of theories, androgyny (Bem, 1974, 1993), status characteristics theory (Ridgeway, 1992), social role theory (Eagly and Karau, 2002), and expectation states theory (Berger et al., 1985), incorporate gender difference in a cross-cultural setting. Thus, future research should enhance scholarship on upwards leadership by incorporating gender and the cultural aspects of leadership construction and effectiveness.

Lastly, a timely exploration for future research is to explain the dynamics of culture and gender in racial diversity (Brathwaite, 2018). Institutional racism against minority groups prompts the underrepresentation of minorities in leadership positions (Bradbury, 2013; Livingston, 2018). Despite the best efforts of organizations to employ a diverse workforce, the drive to boost diversity fails to make progress (Livingston, 2018). Fowler (2020) highlights the facilitators and barriers to leadership in structural racism and gendered contexts. The author proposes that future research should investigate the critical issues pertaining to upward leadership considering racial diversity and the stereotypic notions against the minorities, which could hamper upward leadership in minority groups.

## Conclusion

The proposed theoretical framework explained upward influence and leadership construction from the resource-dependence lens. Dependence relationships, both power-imbalanced and embedded, emerge between employees and managers as they depend on each other for tangible and intangible resources. Leadership is thus co-created by employees and managers in power-imbalanced and embedded relationships when one member claims the leader's identity and the other grants it. Team (specialist vs. uniform) and organizational (mechanistic vs. organic) boundaries were discussed, enabling novel avenues for future research to study the upward influence processes in organizations based on the interplay of dependence, power, resources, and social influence.

## Author Contributions

The author confirms being the sole contributor of this work and has approved it for publication.

## Conflict of Interest

The author declares that the research was conducted in the absence of any commercial or financial relationships that could be construed as a potential conflict of interest.

## Publisher's Note

All claims expressed in this article are solely those of the authors and do not necessarily represent those of their affiliated organizations, or those of the publisher, the editors and the reviewers. Any product that may be evaluated in this article, or claim that may be made by its manufacturer, is not guaranteed or endorsed by the publisher.

## Footnotes

^1^Specialization in tasks here should not be confused by a specialist team. Specialization of tasks refers to classical management approach, in which tasks are divided in such a way that tasks can be performed independently by the workers.

^2^Such cases are evident in the work of Blau (1955) and Crozier (1964) as power struggles between expert subordinates and their superior, for example, the maintenance workers and the middle managers.

^3^Unit, here, refers to the level, for example, individual, dyad, team, organization, etc.
